# The classic EDCs, phthalate esters and organochlorines, in relation to abnormal sperm quality: a systematic review with meta-analysis

**DOI:** 10.1038/srep19982

**Published:** 2016-01-25

**Authors:** Chao Wang, Lu Yang, Shu Wang, Zhan Zhang, Yongquan Yu, Meilin Wang, Meghan Cromie, Weimin Gao, Shou-Lin Wang

**Affiliations:** 1 State Key Lab of Reproductive Medicine, Institute of Toxicology, Nanjing Medical University, 140 Hanzhong Rd., Nanjing 210029, P. R. China; 2 Key Lab of Modern Toxicology of Ministry of Education, School of Public Health, Nanjing Medical University, 101 Longmian Avenue, Nanjing 211166, P.R.China; 3 Kangda Medical College, Nanjing Medical University, 101 Longmian Avenue, Nanjing 211166, P.R. China; 4 Department of Environmental Toxicology, The Institute of Environmental and Human Health, Texas Tech University, 1207 Gilbert Drive, Lubbock, TX 79416, USA

## Abstract

The association between endocrine disrupting chemicals (EDCs) and human sperm quality is controversial due to the inconsistent literature findings, therefore, a systematic review with meta-analysis was performed. Through the literature search and selection based on inclusion criteria, a total of 9 studies (7 cross-sectional, 1 case-control, and 1 pilot study) were analyzed for classic EDCs (5 studies for phthalate esters and 4 studies for organochlorines). Funnel plots revealed a symmetrical distribution with no evidence of publication bias (Begg’s test: intercept = 0.40; p = 0.692). The summary odds ratios (OR) of human sperm quality associated with the classic EDCs was 1.67 (95% CI: 1.31–2.02). After stratification by specific chemical class, consistent increases in the risk of abnormal sperm quality were found in phthalate ester group (OR = 1.52; 95% CI: 1.09–1.95) and organochlorine group (OR = 1.98; 95% CI: 1.34–2.62). Additionally, identification of official data, and a comprehensive review of the mechanisms were performed, and better elucidated the increased risk of these classic EDCs on abnormal sperm quality. The present systematic review and meta-analysis helps to identify the impact of classic EDCs on human sperm quality. However, it still highlights the need for additional epidemiological studies in a larger variety of geographic locations.

Numerous natural and synthetic chemicals have been reported to disrupt the normal function of the endocrine system, and subsequently produce adverse developmental, reproductive, neurological, cardiovascular, metabolic, and immune effects in humans. These chemicals are often classified as endocrine-disrupting chemicals (EDCs), which include both natural and synthetic chemicals. Examples of synthetic chemicals include pharmaceutical agents, pesticides, diethylstilbestrol (DES), dioxin and dioxin-like compounds, polychlorinated biphenyls (PCBs), and components of plastics such as bisphenol A (BPA) and phthalates. EDCs from natural chemicals can include phytoestrogens (*e.g.* genistein and coumestrol), which are found in human and animal food^1^. EDCs are found in many daily products including plastic bottles, metal food cans, detergents, flame retardants, food additives, toys, cosmetics, and pesticides. Therefore, there are many ways in which people can be occupationally and even environmentally exposed to these exogenous compounds, including occupational exposure and general environmental exposure *via* ingestion, inhalation, and skin.

Classic EDCs such as phthalate esters and organochlorines are derived mainly from domestic and industrial effluents, solid waste disposal sites, and agricultural or urban runoff^2^. Previous studies reported that phthalate esters and organochlorines were associated with a wide range of adverse health effects including male and female reproductive problems, obesity, diabetes, and thyroid effects^3^,^4^,^5^. Phthalates are widely used as plasticizers for PVC and other plastics, and they are also used in some cosmetics, paints, and lubricants. Dibutyl-phthalate (DBP), di(2-ethylhexyl)-phthalate (DEHP), and dimethyl-phthalate (DMP) are the most commonly utilized phthalates^6^. PCBs are a class of synthetic, persistent, lipophilic, and halogenated aromatic hydrocarbon mixtures of 200 or more congeners. They were banned in the late 1970s due to their lipophilicity, bioaccumulation ability, and stability. However, PCBs are still globally detected in the air, water, soil, sediment, fish, wildlife, and human adipose tissue, milk, and serum^7^. Organochlorine pesticides (*e.g.* DDT and DDE) are similar to PCBs in that they have been banned in Western countries. However, organochlorine pesticides are still used in some developing countries, and people could be exposed through the environment or even the food chain^8^.

Globally, approximately 15% of heterosexual couples suffer from infertility, half of which, are a result of male reproductive dysfunction due to malformations of the reproductive tract, infections, genetic causes, and chemical exposure^9^,^10^. Epidemiological evidence reveals that male reproductive health has been declining in the last decades, particularly in Western nations. For example, sperm counts in Western countries appear to have declined by half in the past 50 years, which seems likely to play roles in the recent decline in fertility rates^11^. Travison *et al.* recently reported declining levels of testosterone in US men of 1% per year, and the same rate of decline was seen in sperm concentrations^12^. Possible exposure to EDCs may play a role in the temporal downward trend in sperm quality and testosterone levels among adult male populations^13^.

Animal toxicological studies^14^, cellular experiments^15^, and human studies^16^ have demonstrated that some EDCs could exert adverse effects on the male reproductive system *via* sexual hormone and related receptor signaling pathways^1^. A recent study found that BPA affected the hypothalamic-pituitary-testicular axis through modulating hormone (*e.g.* luteinizing hormone (LH), follicle stimulating hormone (FSH), androgen and estrogen synthesis, expression, and function of respective receptors (*e.g.* estrogen receptor (ER), androgen receptor (AR)), which resulted in sperm alterations^17^. *p,p*′-DDE and DDT cause reductions in sperm concentration, morphology, and impair sperm motility^18^,^19^, and similar findings have been observed in other persistent environmental contaminants such as PCBs^20^,^21^. A large study on male partners of subfertile couples found associations between monobutyl phthalate (MBP; the hydrolytic metabolite of dibutyl phthalate) and abnormal sperm motility and sperm concentration. A dose-response relationship was found between monobenzyl phthalate (MBzP, the primary hydrolytic metabolite of butylbenzylphthalate) and low sperm concentration^22^. Overall, the decreasing trend in male fertility in terms of sperm counts and sperm quality has been correlated to EDC exposure in some instances^23^.

A substantial body of evidence has accumulated in recent years indicating the various adverse health effects of EDCs on male reproductive health. However, there are studies suggesting that there is not enough evidence to support the association between environmental or occupational exposure of EDCs and the adverse effects on male reproduction. An European Union-supported study failed to show any correlation between post-natal exposure levels of persistent organohalogen pollutants ((*e.g.* CB-153, *p,p*′-DDE) and fertility^24^. In addition, a small cross-sectional occupational study in South Africa showed few significant associations between DDT exposure and reproductive outcomes^25^. Studies in North America and in Europe have also shown no evidence for major effects of PCB and *p,p´*-DDE on sperm parameters or fertility^16^. A study in Sweden of young males in the military showed no relationship between MBP or MBzP with any of the sperm parameters^26^. MEHP was also not associated with abnormal sperm parameters, but men in the highest quartile of MEP exposure had fewer motile sperm and more immotile sperm than those in the lowest quartile^26^. Moreover, most studies, including several large prospective studies, found no evidence that occupational exposure to pesticides had any major impact in Western countries^27^,^28^.

In summary, the epidemiological data on sperm quality in relation to EDC exposure remains limited and inconsistent. Therefore, we aimed to analyze the association between the exposure to classic EDCs and male sperm quality through a systematic review with a meta-analysis. In addition, a comprehensive review concerning the mechanisms of EDC-induced male reproductive dysfunction was presented. This systematic review with meta-analysis will help to provide a better understanding of the impact of EDCs on male reproductive health, and possible mechanisms as well.

## Results

### Study characteristics

**Figure 1:**
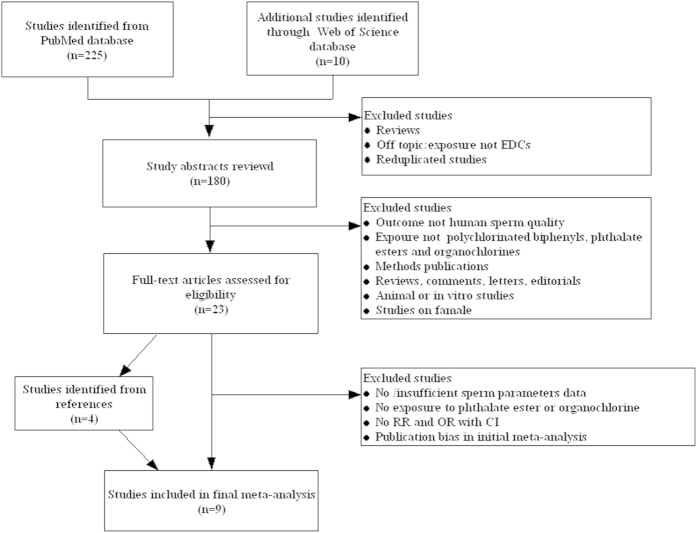
Flow diagram of the study selection process. EDCs: endocrine disrupting chemicals; RR: relative risks; OR: odds ratios; CI: confidence intervals.

**Figure 2:**
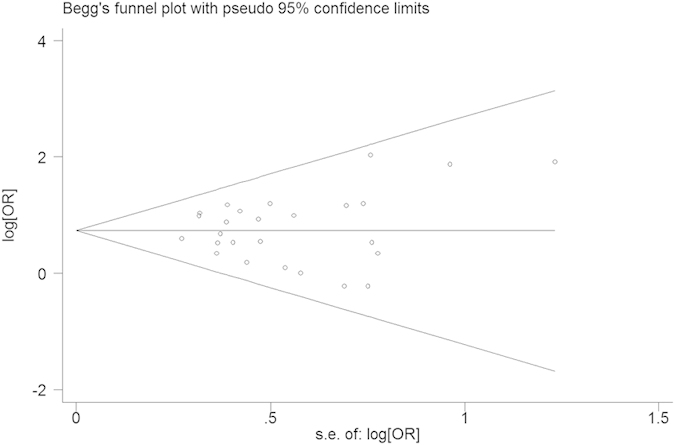
Funnel plot for the detection of publication bias on EDCs and human sperm quality. Each dot represents a separate study for the indicated association. Location outside the delineated triangle (pseudo 95% confidence limits) suggests a publication bias.

Through the literature search and selection based on inclusion criteria, 9 articles were identified by reviewing potentially relevant articles (Fig. 1), including seven cross-sectional, one case-control, and one pilot study for the present meta-analysis. The characteristics of the selected studies are shown in Table 1. Seven studies were conducted in the US^16^,^21^,^22^,^29^,^30^,^31^,^32^ and there were two in China^33^,^34^. The literature was divided into two categories after stratification by specific chemical class; one class for phthalate esters and the other class for organochlorines. Funnel plots revealed a symmetrical distribution with no evidence of publication bias (Begg’s test, intercept = 0.40; *p* = 0.692) (Fig. 2). There was one case-control study with a total of 25 male infertility cases, seven cross-sectional studies involving 2016 male partners of subfertile couples, and one pilot study involving 45 male partners of subfertile couples (Table 1).

### Meta-analysis

**Figure 3:**
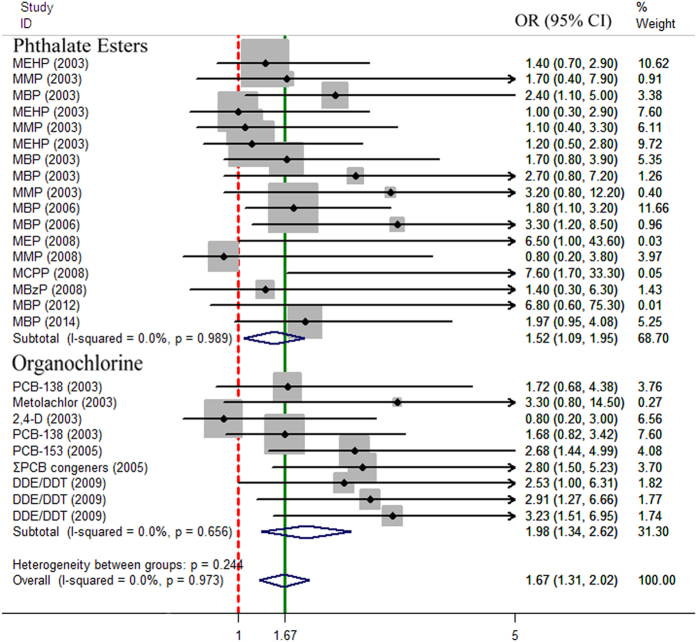
Forest plot of phthalate ester, organochlorine and human sperm quality. Studies are plotted according to the name of chemicals and followed by the publication year in parentheses. Horizontal lines represent 95% CI, the arrows mean the values exceed the length of abscissa. Each square represents the OR point estimate and its size is proportional to the weight of the study. The diamond (unbroken line) represents the overall summary estimate with CI given by its width. The broken vertical line is at the null value (OR = 1). CI, confidence interval; OR, odds ratio.

Nine studies contributing a total of 26 odds ratio (OR) estimators met the inclusion criteria and were taken into consideration. The summary OR of abnormal sperm quality associated with exposure to classic EDCs was 1.67 (95% CI: 1.31–2.02) by both fixed- and random-effects models (Fig. 3). The heterogeneity Q statistic was 13.50 (*p* = 0.973, *p* > 0.05) and the I^2^ was 0.00%, indicating no statistical evidence for heterogeneity. Furthermore, in the subgroup analysis based on specific chemical class, the overall association between phthalate ester and abnormal sperm quality was statistically significant for the five studies (OR: 1.52, 95% CI: 1.09–1.95, *p* = 0.989), and an obvious increase in the risk of abnormal sperm quality was found in the organochlorine group (OR = 1.98, 95% CI: 1.34–2.62, *p* = 0.656). In addition, a forest plot of the 9 studies displayed the weights applied in each study for the overall meta-analysis (Fig. 3). The class of phthalate esters and organochlorine pesticides contributed 68.70% and 31.30% of the total weight, respectively. No single study contributed more than 30% of the total weight, thus, the overall estimate risk contributed the largest number of cases, which was not directly affected by a single study with a methodological difference.

### Review of official data

Present results suggested that exposure to organochlorine and phthalate esters appeared to be associated with an increased risk of abnormal sperm quality. To validate the findings we performed an additional systematic review using official research data, reports, and relevant literature reviews on the relationship between classical EDCs and sperm quality.

#### (1) PCBs and sperm quality

As shown in Table 2, PCBs and their congeners (PCB 153, PCB 138) were associated with abnormal sperm motility and morphology in humans, and PCB 132, PCB 118, PCB 77, PCB 126 induced a reduction in sperm number and daily sperm production in rats. Such relationships have been consistently reported across studies performed in different countries. For example, an epidemiology study from the United States reported that high-doses of PCBs from accidental food contamination presented a dose-response relationship with sperm motility (ORs per tertile of PCB 138, 1.00, 1.68, 2.35) and morphology (1.00, 1.36, 2.53)^35^. Environmental exposure to lower doses of PCBs also supported an association with reduced sperm quality, specifically sperm motility^36^. Richthoff *et al.*^37^ found a weak, but statistically significant negative correlation between PCB 153 levels and both the ratio of testosterone:SHBG (sex hormone-binding globulin) (r = −0.25), and sperm motility (r = −0.13). Among 195 Swedish fishermen, the subjects in the highest quintile of PCB 153 exposure (>328 ng/g lipid) tended to have decreased sperm motility compared with those in the lowest quintile (<113 ng/g lipid), but this association was weak after age-adjusted (*p* = 0.08)^20^.

#### (2) Organochlorine pesticides and sperm quality

Organochlorine pesticides may affect human sperm abnormalities including sperm count, morphology, motility, and seminal volume (Table 3). Two recent studies found associations between pesticide representatives and reduced sperm quality in the general population^38^,^39^. An increased risk for poor sperm quality was found in relation to urinary concentrations of several pesticides such as alachlor, 2-isopropoxy-4-methyl-pyrimidinol, atrazine, 1-naphthol, and 3,5,6-trichloro-2-pyridinol^38^. A cross-sectional study in Limpopo, South Africa showed that serum DDT derivatives (six compounds) induced morphology scores 84% below either the World Health Organization (WHO) or the Tygerberg criteria^40^. Another cross-sectional study in Limpopo that found that higher plasma DDE was associated with reduced sperm motility, reduced ejaculate volume, and oligozoospermia. Both DDT and DDE were significantly associated with asthenozoospermia^18^. A study in Mexico demonstrated that higher plasma DDE levels were associated with the decreased percentage of motile sperm and the increased percentage of sperm with morphological tail defects^19^. Another study from infertility clinics in Michigan, USA indicated significant associations between *p,p*′-DDE serum levels and reduced sperm concentration, motility, and morphology (depending on DNA polymorphisms), and the risk for low sperm concentration was significantly increased by high DDE and DDT serum concentrations^31^. Additionally, the increased *p,p*′-DDE in serum was associated with a moderate, but significant increase in chromatin defects in the sperm of 209 young men from DDT-sprayed dwellings in South Africa^41^. Ayotte *et al.* found that *p,p′*-DDE concentration was inversely correlated to both semen volume (r = −0.47) and sperm count (r = −0.42)^42^.

#### (3) Phthalates and sperm quality

Phthalate esters such as DBP, DEHP, and DMP are commonly utilized in industry and urinary concentrations of phthalate metabolites, such as MEP, MEHP, MBzP, MBP, MMP, MCPP, are common biomonitoring approaches. Studies that explored the effects of phthalates on male reproductive health are presented in Table 4. The results show that phthalates might be associated with decreased sperm concentration, motility, morphology, and sperm DNA damage in humans or rats. In Andhra Pradesh, India, an epidemiological study showed that phthalates might be instrumental in the deterioration of semen quality in infertile men, especially increased abnormal sperm morphology^2^. In the general population of young Swedish males, Jonsson *et al.*^26^ suggested that subjects within the highest quartile for MEP had fewer motile sperm and more immotile sperms. Hauser^43^ from the National Institute of Environmental Health Sciences (NIEHS) reported MEP and MEHP were associated with increased sperm DNA damage. Another study in Massachusetts General Hospital, demonstrated that urinary MEP, at environmental levels, was associated with the increased DNA damage in sperm^44^.

## Discussion

This systematic review with meta-analysis extracted the estimates of association of two classic EDCs, phthalate esters and organochlorines, from nine independent studies from two countries (majority in North America), and the results showed consistent evidence of positive associations of these classic EDCs with abnormal sperm quality (Fig. 3). The heterogeneity Q statistic was 13.50 (*p* = 0.973, *p* > 0.05) and I^2^ was 0.00%, indicating no statistical evidence for heterogeneity among the selected studies. Begg’s funnel plot and Egger’s test were usually performed to assess the publication bias of literature. Particularly, the Egger’s test was used to provide statistical evidence of funnel plot symmetry. Our results showed no evidence of obvious asymmetry in the shape of the funnel plot, and no publication bias (Egger’s test*, p* = 0.691), indicating that the present results in meta-analysis were reliable.

Among the studies in this meta-analysis (Table 1), Hauser *et al.*^30^ found that PCB-153 in relation to sperm motility, and there were dose-response and inverse relationships among PCB-138 and sperm motility and morphology in their another study^16^. Sperm motility may be the target effect of PCBs. High DDE-DDT exposure adversely affected some sperm parameters (sperm count, motility and morphology)^31^. However, there was no significant association between organochlorines and semen quality^32^. Phthalate esters seem to show the weak adverse effects on sperm quality. Hauser *et al.* reported near significant (highest quartile of MBzP *vs* low sperm concentration, *p* = 0.13) or significant associations between urinary levels of monobutyl phthalate and low sperm counts (*p* = 0.02) and/or motility (*p* = 0.04)^21^, and similar trends were found for additional phthalate metabolites (monobenzyl phthalate) in other studies^22^,^29^,^33^. But a study from general population in Chongqing, China, showed that exposure to the environmental level of phthalate had weak adverse effects on the sperm concentration^34^.

In addition, through the analysis of official research data, reports, and relevant literature, we found that occupational and environmental exposure to the aforementioned EDCs are closely related to male reproductive damage (Table 2, Table 3, Table 4). For example, both animal studies and epidemiological evidence supported an inverse association between PCBs and sperm quality^35^,^37^,^45^,^46^,^47^,^48^,^49^,^50^. Serum levels of DDE/DDT were associated with reduced sperm concentration, motility, and morphology^18^,^19^,^25^,^38^,^42^,^51^. Still, there were significant associations between phthalate esters and poor sperm quality^2^,^26^,^43^,^44^,^52^. Notably, interactions between MBP and MBzP with the PCB-153 congener, in relation to sperm motility were found^30^. Despite these findings, some studies have shown no evidence that supports these negative effects in sperm parameters or fertility in *p,p*′-DDE^53^,^54^, MMP, and MBzP^29^. Therefore, due to the limited amount of literature in the present meta-analysis, and inconsistent findings among studies related to EDC exposure and human sperm quality, it further highlights the need for additional epidemiological studies in a large variety of geographic locations.

**Figure 4:**
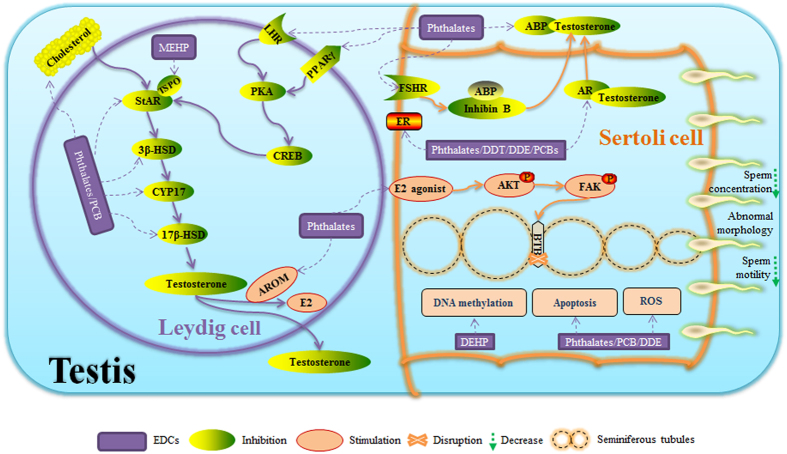
A schematic mechanism on the effects of phthalate esters and organochlorines on testosterone and sperm quality. A. Steroidogenesis. EDCs can inhibit the synthesis of testosterone through direct pathways including cholesterol, StAR, 3β-HSD, CYP 17, and 17β-HSD, or indirect pathways including the binding of LH to LH receptor and PPARγ, PKA, and StAR, or through FSH receptor, or the binding of testosterone to ABP or AR. In addition, EDCs can increase the AROM activity, which converts testosterone to estrogen, resulting in the decrease in testosterone. B. Spermatogenesis. EDCs may affect spermatogenesis through the apoptosis of spermatocytes, ROS production, or disrupting BTB integrity *via* the activation of PI3K/FAK or PI3K/Akt and MAPK/ERK signaling pathways. C. DNA damage and DNA methylation. CpG islands may be possible mechanisms of EDC-induced testicular toxicity and sperm quality. LHR, luteinizing hormone receptor; PPARγ, peroxisome proliferator activated receptor gamma; PKA, protein kinase A; CREB, cAMP response element; StAR, steroidogenic acute regulatory protein; TSPO, translocator protein; 3β-HSD, 3β hydroxysteroid; CYP 17, Cytochrome P450 17; 17β-HSD, 17β hydroxysteroid; AROM, cytochrome P450 aromatase; ROS, reactive oxygen species; AR, androgen receptor; ER, estrogen receptor; FSH, follicle stimulating hormone; ABP, androgen binding protein; PI3K, phosphatidylinositol 3 kinase; FAK, focal adhesion kinase; MAPK, mitogen activated protein kinase; ERK, extracellular regulated protein kinases; BTB, blood–testis barrier.

To address these concerns, we summarized the mechanism of action for organochlorines and phthalates on sperm quality from several *in vivo* and *in vitro* studies (Fig. 4). Animal, clinical and epidemiological studies have demonstrated that exposure to EDCs disrupts male reproductive health, traditionally through male steroidogenesis to disrupt spermatogenesis. Phthalates and its metabolite, DEHP, were observed to exert an effect on Leydig or Sertoli cell structure and functions through the activation of peroxisome proliferator activated receptors (PPARs). DEHP also affected the binding of LH to G-protein coupled LH receptors, thereby influencing steroid hormone biosynthesis in fetal rat testes^55^, and DEHP was found to inhibit testosterone production resulting in the dysfunction of StAR, 3β-HSD, CYP17, and 17β-HSD. In addition, phthalates have been shown to disrupt the patterns of gene expression that regulate cholesterol and lipid homeostasis or insulin signaling, resulting in lower testosterone synthesis^56^. Still, phthalates could decrease testosterone through the induction of cytochrome P450 aromatase (AROM), which converts testosterone to estrogen in cultured rat cerebellar granule cells^56^. Similarily, PCB 153 and Aroclor 1254 promoted the down-regulation of StAR, 3β-HSD, CYP 17, and 17β-HSD in 14-year-old boys from a birth cohort in the Faroe Islands^57^. Prenatal PCB exposure was associated with lower serum concentrations of LH and testosterone in rats^58^. Select phthalate monoesters may interfere with the ability of Sertoli cells to respond to their normal endogenous ligand, FSH^59^, thereby affecting the downstream secretion of androgen binding protein (ABP) which could induce reproductive dysfunction.

The spermatogenic process is usually regarded as both a source and a target of reactive oxygen species (ROS). EDCs could interfere with testicular functions by breaking the dynamic balance of this antioxidant system, and subsequently affect sperm quality. Phthalates, mainly DEHP, DBP, DEP, MEHHP, MEOHP, and MEHP, are associated with specific events in spermatogenesis including the induction of ROS, lipid peroxidation, and apoptosis of spermatocytes in mice^60^. In neonatal Sertoli cell/gonocyte coculture system, MEHP was found to induce germ cell apoptosis through the Fas/FasL pathway^61^. PCB 132 impaired sperm function and altered testicular apoptosis-related gene expression through the generation of ROS, activation of caspase-3 and -9, and down-regulation of Fas, Bax, bcl-2, and p53 genes in rat offspring^14^. In other studies, PCB 153 and *p,p′*-DDE were found to induce ROS and cellular apoptosis in a Sertoli cell/gonocyte co-culture system^62^,^63^. Moreover, exogenous estrogen (*e.g.* EDCs) and endogenous estrogen can induce germ cell apoptosis and blood-testis barrier (BTB) dysfunction via PI3K/FAK or PI3K/AKT and MAPK/ERK signaling pathways in mice^64^. For example, phthalates have the ability to directly damage the BTB integrity by allowing the BTB to “open” and disrupt spermatogenesis in rats^65^. Notably, local biosynthesis of estrogen and androgen occurs in the testis when aromatase is expressed in Leydig cells and some populations of germ cells^66^. EDCs can interfere with the binding of the hormone and the receptor, and disrupt the normal process of spermatogenesis. Some organochlorines, such as *p,p*′-DDE, and other PCBs, are regarded as ER antagonists, antiestrogens, or AR antagonists^67^,^68^. Phthalates, DDT, and DDT metabolites (*o,p*′-DDT, and *p,p*′-DDE) can inhibit endogenous ligands from binding estrogen and androgen receptors^69^. PCBs can disrupt estrogen receptor function by mimicking the natural ligand and acting as an agonist^70^. Additionally, human semen MMP, MEP, and urinary phthalate metabolites (MBzP, MBP, MEHP, and MEP) were associated with increased sperm DNA damage and sperm aneuploidy^71^,^72^. A majority of organophosphate pesticides, including PCB, DDT, and *p,p*′-DDE, affect the male reproductive system by inducing sperm DNA damage^73^. DEHP can induce changes in DNA methylation within CpG islands, resulting in testicular toxicity^74^.

Current evidence has shown limitations in meta-analysis regarding the relationship between EDCs and human sperm quality. First, studies with small sample sizes may not adequately explore the potential exposure-response relationships. Secondly, differences exist between epidemiological studies, including differences in sample size, study design, study populations, life stage, data analysis approaches, strategies for exposure, and endpoints of effects. Thirdly, some of the extracted effects were unadjusted and a more precise analysis, perhaps through multivariable analysis instead of univariate, should be conducted from all the data. Finally, there are limited inherent epidemiological studies that evaluate the toxicity of multiple chemicals and to our knowledge, humans are exposed to a mixture of chemicals rather than a single chemical.

Nonetheless, our systematic review with meta-analysis, together with the review of possible mechanisms, provides a better explanation of the impact of classic EDCs on sperm quality. However, future research is needed to examine the following: (1) the biomarker of testis function and human fertility should be well defined in an epidemiology study, and the analysis of sperm quality parameters need to be normalized; (2) the size of adequate samples, occupational exposure to specific EDCs, longitudinal instead of cross-sectional studies, and multi-center studies need to be conducted; (3) due to potential interactions between different EDCs on sperm quality, co-exposure to mixtures of EDCs, as well as their interactions or combined effects should be investigated; (4) for a better understanding of classic EDC-induced abnormal sperm quality, mechanism studies should be focused on low-dose, long-term, and co-exposure; and (5) both human studies and animal experiments are needed on transgenerational effects (*e.g.* DNA methylation) of EDCs because epigenetic effects as a result of EDC exposure can subsequently change the sperm quality of future generations.

## Methods

### Literature search

We performed a systematic electronic search on the National Library of Medicine PubMed database and Web of Science database to identify published studies from January 1990 to April 2015. The research question was defined as ‘what are the associations between classic EDCs and sperm quality?’. This question was subsequently broken down to cover specific search terms such as ‘endocrine disrupting chemicals’, ‘male reproductive damage’, ‘infant development’, ‘abnormal development’, ‘malformation’, ‘infertility’, ‘abnormal sperm’, ‘sperm parameter’, ‘asthenospermia’, ‘aspermia’, and ‘oligospermia’. Through searching and examining relevant literature to fill the missing components, each of them was cross-referenced with the following classic EDC terms: ‘bisphenol A’, ‘genistein’, ‘cadmium’, ‘lead’, ‘phthalates’, ‘poly chlorinated biphenyls’, ‘polybrominated diphenyl ethers’, ‘perfluorooctane sulfonates’, ‘topical corticosteroid dependent dermatitis’, ‘pesticide’, ‘DDT’, and ‘DDE’. However, only the term ‘phthalates’, ‘poly chlorinated biphenyls’, ‘DDT’ and ‘DDE’ were finally selected due to the lack of human studies or insufficient data for other EDCs. In addition, we replicated the search in the Web of Science database to identify additional pertinent references. Searches were restricted to human trials with the language restriction of English. All the references of relevant articles were scanned for additional analysis.

### Selection of studies

Studies selected for the meta-analysis met the following inclusion criteria: (1) Written and published in English; (2) Reported results from case-control, cohort, or cross-sectional epidemiology studies; (3) A relative risks (RR) and odds ratios (OR) with confidence intervals (CI) was reported, or could be calculated from provided data; (4) Referred to environmental or occupational exposure to the classic EDCs, phthalate esters and organochlorines.

Studies were excluded from the analysis if they: (1) included subjects that were already included in another more complete or more recent study; (2) did not report original results (reviews, comments, letters, editorials); (3) investigated women studies.

### Data extraction

Each eligible study was classified as pilot, cross-sectional, cohort, or case-control study. We extracted the following information from the full text of each eligible publication: (1) author; (2) publication year; (3) study design (cross-sectional, case-control, or cohort); (4) exposure type; (5) source population for the controls in case-control studies; (6) number of cohort participants or number of cases and controls; (7) experimental results and health outcomes; (8) the name and category of EDCs; (9) effect estimates (RR, OR, and p-value) and CI.

### Meta-analysis

The data was synthesized using both fixed-effects and random-effects models weighting each study by a measure of its precision, the inverse of the estimate variance. Heterogeneity of effects across studies was assessed by the Cochran’s Q statistic^75^ and was deemed significant when P < 0.05. In addition, the coefficient of inconsistency (I^2^) as described by Higgins and Thompson^76^ was also computed to assess heterogeneity. To examine the possibility that publication bias may have affected the results, a funnel plot of the natural logarithm of OR was constructed as the inverse of the variance of the studies, and regression test for the effects of small studies^77^ was used for quantitative assessment of publication bias and funnel plot asymmetry. The data on ORs and 95% CI were entered into the STATA 12.0 statistical package to perform these calculations, and META command was used to calculate a summary OR, 95% CI, and heterogeneity statistics. The META-BIAS command was used to conduct the Begg’s test which is used to diagnose publication bias and approximate to the fact than Egger’s test if the publications are tendentiously deleted^78^.

### Identification of official data

To validate the results from the meta-analysis, we performed a systematic electronic search related to phthalate esters, organochlorines, and sperm quality from the website databases of the U.S Environmental Protection Agency (EPA), WHO, U.S. Centers for Disease Control and Prevention (CDC), National Institutes of Health (NIH), and the NIEHS. These official research data and reports were then summarized in order to support our findings.

### Systematic review on mechanisms from animal experiments and *in vitro* studies

Recently, a large number of experimental animal studies have shown that EDCs have strong reproductive toxicity through steroid hormone synthesis, and possibly alter reproductive hormones to disrupt spermatogenesis. These studies provide new insights about other mechanisms such as oxidative stress, genetic susceptibility, and epigenetic effects. Therefore, a comprehensive review was additionally conducted through animal experiments and *in vitro* studies related to the mechanisms for the effects of phthalate esters and organochlorines on male reproductive damage.

## Additional Information

**How to cite this article**: Wang, C. *et al.* The classic EDCs, phthalate esters and organochlorines, in relation to abnormal sperm quality: a systematic review with meta-analysis. *Sci. Rep.* **6**, 19982; doi: 10.1038/srep19982 (2016).

## Figures and Tables

**Table 1:** Main characteristics of all epidemiological studies on sperm quality and EDCs included in the meta-analysis.

Compounds	Study design	N in Analysis	Specimen	Concentration	Sperm quality	Results	Author (year)
**MBP**	Cross-sectional	232 general men	urine	22.9 μg/g (CR adjusted)	Weak association with low sperm concentration	Sperm concentration (OR = 1.97,95%CI: 0.95,4.08)	Han, X *et al.* (2014)^34^
**MBP**	Cross-sectional	150 individuals recruited from reproductive institute	urine	25.7 ng/mL(CR adjusted)	↓Sperm concentration	Sperm concentration (OR = 6.8,95%CI: 0.6,75.3,*p* = 0.05)	Liu *et al.* (2012)^33^
**MEP**	Pilot	45 male partners of subfertile couples	urine	121.9 ng/mL	↓ Sperm concentration	(OR = 6.5,95% CI: 1.0–43.6, *p* < 0.05)	Wirth *et al.* (2008)^29^
**MBzP**	Pilot	45 male partners of subfertile couples	urine	26.9 ng/mL	No association	(OR = 1.4, 95% CI: 0.3, 6.3)	
**MMP**	Pilot	45 male partners of subfertile couples	urine	1.1 ng/mL	No association	(OR = 0.8, 95% CI: 0.2, 3.8)	
**MCPP**	Pilot	45 male partners of subfertile couples	urine	2.5 ng/mL	↓ Morphology	(OR = 7.6, 95% CI: 1.7–33.3)	
**MBP**	Cross-sectional	463 male partners of subfertile couple	urine	17.3 ng/mL	↓ Sperm concentration, motility	Sperm concentration (OR = 3.3,95%CI: 1.2,8.5) Sperm motility (OR = 1.8,95%CI: 1.1,3.2)	Hauser *et al.* (2006)^21^
**MBP**	Cross-sectional	168 male partners of subfertile couples	urine	16.1 ng/mL	↓ Sperm concentration, motility, morphology	Sperm concentration (OR = 2.7,95%CI: 0.8,7.2) Sperm motility (OR = 2.4,95%CI: 1.1,5.0) Sperm morphology (OR = 1.7,95%CI: 0.8,3.9)	
**MEHP**	Cross-sectional	168 male partners of subfertile couples	urine	7.6 ng/mL	No association	Sperm concentration (OR = 1.0,95%CI: 0.3,2.9) Sperm motility (OR = 1.4,95%CI: 0.7.2.9) Sperm morphology (OR = 1.2,95%CI: 0.5,2.8)	Duty *et al.* (2003a)^22^
**MMP**	Cross-sectional	168 male partners of subfertile couples	urine	7.5 ng/mL	↓ Morphology	Sperm concentration (OR = 1.7,95%CI: 0.4,7.9) Sperm motility (OR = 1.1,95%CI: 0.4,3.3) Sperm morphology (OR = 3.2,95%CI: 0.8,12.2)	
**PCB-153**	Cross-sectional	303 male partners of subfertile couple	serum	43 ng/g lipids	↓ Motility	OR = 2.68 (95%CI: 1.44–4.99)	Hauser *et al.* (2005)^30^
**ΣPCB congeners**	Cross-sectional	303 male partners of subfertile couple	serum	223 ng/g lipids	↓ Motility	OR = 2.80 (95%CI:1.50–5.23)	
**PCB-138**	Cross-sectional	212 male partners of subfertile couples	serum	33.6 ng/g lipids	↓ Morphology ↓ Motility	Sperm motility (OR = 1.68,95%CI: 0.82,3.42,*p* = 0.03) Sperm morphology (OR = 1.36,95%CI: 0.57,3.22*,p* = 0.04)	Hauser *et al.* (2003)^16^
**DDE/DDT**	Cross-sectional	336 male partners of couples presenting to infertility clinics	serum	0.05 μg/g lipid (*p,p′*-DDT), 0.29 μg/g lipid (*p,p′*-DDE)	Low sperm count, motility and abnormal morphology	Sperm count (OR = 2.53; 95% CI = 1.0–6.31), Motility (OR = 2.91; 95% CI = 1.27–6.66), Morphology (OR = 3.23; 95% CI = 1.51–6.95)	Messaros *et al.* (2009)^31^
**Metolachlor**	Case-control	25 cases and 25 controls	urine	0.48 μg/g (CR adjusted)	No association	OR = 3.3 (95% CI:0.8–14.5)	Swan *et al.* (2006)^32^
**2,4-D**	Case-control	25 cases and 25 controls	urine	0.56 μg/g (CR adjusted)	No association	OR = 0.8 (95% CI:0.2–3.0)	

Abbreviations: OR, odds ratio; CI, confidence interval; CR: creatinine.

**Table 2:** Effects of polychorinated biphenyls on sperm quality observed in humans and animals.

Compounds	Species	Observation	References
**PCBs**	Human	↓ Sperm motility	Hauser *et al.* (2006)^36^
**PCB 153**	Human	↓ Sperm motility, weak association	Rignell-Hydbom *et al.* (2004)^20^
**PCB 138**	Human	↓ Sperm motility, ↓ morphology	Hsu *et al.* (2003)^35^
**PCB 153**	Human	↓ Sperm motility	Richthoff *et al.* (2003)^37^
**PCB 132**	Rat	↓Sperm number, ↓motile sperm count	Hsu *et al.* (2007)^14^
**PCB 118**	Rat	↓ Motile sperm count, ↑abnormal sperm	Kuriyama & Chahoud (2004)^47^
**PCB 77**	Rat	↓ Daily sperm production, ↑abnormal sperm	Faqi *et al.* (1998)^48^

**Table 3:** Effects of organochlorine pesticides on sperm quality observed in the male reproductive system of humans.

Compounds	Species	Observation	References
**DDE,DDT**	Human	↓Sperm count, ↓ sperm motility, ↑abnormal sperm morphology	Messaros *et al.* (2009)^31^
**DDE,DDT**	Human	↑Sperm chromatin integrity	De Jager *et al.* (2009)^41^
**β-HCH,λ-HCH,** ***p,p***′**-DDE,** ***p,p***′**-DDD**	Human	↓Sperm quality	Pant *et al.* (2007)^51^
***p,p***′**-DDT,** ***p,p***′**-DDE**	Human	Asthenozoospermia	Aneck-Hahn *et al.* (2007)^18^
**DDE**	Human	↓ Motile sperm	De Jager *et al.* (2006)^19^
***p,p***′**-DDE**	Human	Weak association with sperm motility	Hauser *et al.* (2003)^16^
**Pesticides**	Human	↓Sperm quality	Swan *et al.* (2003)^38^
***p,p***′**-DDE**	Human	↓Seminal volume, ↓Sperm count	Ayotte *et al.* (2001)^42^

**Table 4:** Effects of phthalates on sperm quality observed in humans and animals.

Compounds	Species	Observation	References
**MEP,MEHP**	Human	↑Sperm DNA damage	Hauser *et al.* (2007)^43^
**MBP, MBzP**	Human	↓Sperm concentration, ↓sperm motility	Hauser *et al.* (2006)^21^
**MEP**	Human	↓Sperm motility	Jonsson *et al.* (2005)^26^
**MBP, MBzP**	Human	↓Sperm concentration, ↓sperm motility	Duty *et al.* (2003a)^22^
**MEP**	Human	↑Sperm DNA damage	Duty *et al.* (2003b)^44^
**MEP,MCPP**	Human	↓Sperm concentration, ↓ morphology	Wirth *et al.* (2008)^29^
**Phthalates**	Human	↓Sperm morphology	Rozati *et al.* (2008)^2^
**DEHP**	Rat	↓Daily sperm production	Andrade *et al.* (2006)^52^
